# On the Frequency Response of Nanostructured Thermoacoustic Loudspeakers

**DOI:** 10.3390/nano8100833

**Published:** 2018-10-14

**Authors:** Paolo La Torraca, Marco Bobinger, Maurizio Servadio, Paolo Pavan, Markus Becherer, Paolo Lugli, Luca Larcher

**Affiliations:** 1 Department of Engineering "Enzo Ferrari", University of Modena and Reggio Emilia, 41125 Modena, Italy; paolo.pavan@unimore.it; 2 Chair of Nanoelectrics, Technical University of Munich, 80333 Munich, Germany; markus.becherer@tum.de; 3 ASK Industries S.p.A., 60037 Monte San Vito (AN), Italy; servadiom@askgroup.it; 4 Faculty of Science and Technology, Free University of Bozen-Bolzano, 39100 Bolzano, Italy; paolo.lugli@unibz.it; 5 Department of Science and Methods for Engineering, University of Modena and Reggio Emilia, 42122 Reggio Emilia, Italy; luca.larcher@unimore.it

**Keywords:** thermoacoustic, loudspeaker, silver nanowire, nanometric gold film, thermal response, acoustic response, heat distribution, thermal effusivity

## Abstract

In this work, we investigate the thermal and acoustic frequency responses of nanostructured thermoacoustic loudspeakers. An opposite frequency dependence of thermal and acoustic responses was found independently of the device substrate (Kapton and glass) and the nanometric active film (silver nanowires and nm-thick metal films). The experimental results are interpreted with the support of a comprehensive electro-thermo-acoustic model, allowing for the separation of the purely thermal effects from the proper thermoacoustic (TA) transduction. The thermal interactions causing the reported opposite trends are understood, providing useful insights for the further development of the TA loudspeaker technology.

## 1. Introduction

Thermoacoustic (TA) loudspeakers (or thermophones) are electroacoustic transducers exploiting the thermoacoustic effect to generate sound. This technology has long been known [1], but the lack of conductive, low heat capacity materials, required to support an efficient transduction, prevented its further development.

The recent advancements in the synthesis of nanostructured materials enabled the fabrication of thin, conductive and low heat capacity layers, which have been successfully used for the design and fabrication of many types of thermoacoustic loudspeakers. Working TA loudspeakers have been fabricated using carbon nanotubes (CNT) [2,3], silver nanowires (AgNW) [4], poly(3,4-ethylenedioxythiophene):poly(styrenesulfonic) (PEDOT:PSS) [5], indium-tin oxide (ITO) [6], gold nanowires (AuNW) [7], graphene foam [8], graphene [9], and copper nanowires (CuNW) [10].

Nevertheless, most of the latest studies focused on the evaluation of the performance enabled by the new materials, often overlooking the physical phenomena underlying TA sound generation. While the full electro-acoustic transduction of TA loudspeakers is widely documented in the literature [1,11,12,13,14,15,16,17], the thermal response of TA loudspeakers to the applied electrical stimuli and its role in determining the acoustic response were investigated superficially in the past.

Only recently, the full electro-acoustic transduction of AgNW TA loudspeakers has been investigated considering the atomic electro-thermal and thermoacoustic transductions separately [18]. The thermal and acoustic responses of the AgNW TA loudspeakers have been modeled and characterized in frequency using a high-speed IR thermographic camera and a precision microphone, respectively, providing experimental evidences of their different trend.

Despite the intuitive connection between the surface temperature modulation and the sound generation, the TA loudspeaker thermal response is characterized by a “low-pass” dynamic [18], which contrasts with the “high-pass” behavior of the acoustic response [14]. The causes of this phenomena, lying in the thermal interactions involved in the TA sound generation, have never been thoroughly studied and are quantitatively investigated in this paper.

For this purpose, the thermal and the acoustic frequency responses of TA loudspeakers with different substrates (Kapton, glass) and nanometric active films (silver nanowires, thin gold film) are interpreted using a physical model, in order to gain insights into the key processes governing both the electro-thermal and the thermoacoustic transductions. 

## 2. Materials and Methods

### 2.1. Thermoacoustic Loudspeaker Fabrication

**Figure 1:**
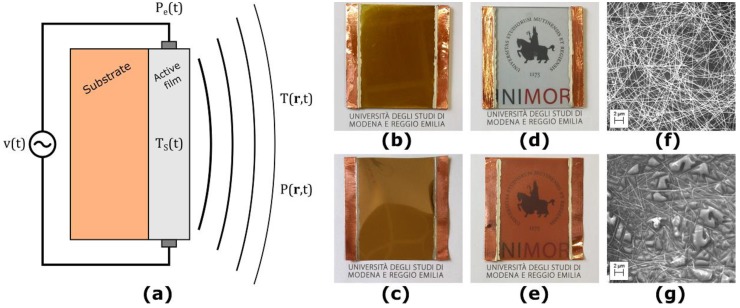
The considered thermoacoustic (TA) loudspeakers. (**a**) Structure of the TA loudspeakers; (**b**) sample with gold active film on glass substrate; (**c**) sample with gold active film on Kapton substrate; (**d**) sample with silver nanowires (AgNW) active film on glass substrate; (**e**) sample with AgNW active film on Kapton substrate; (**f**) SEM-image of the AgNW active film on glass substrate; (**g**) SEM-image of the AgNW active film on Kapton substrate.

The considered TA loudspeaker is composed of an electrically insulating substrate, upon which a thin, nanostructured active film made of conductive material is deposited. The structure of such devices is depicted in Figure 1a. We considered TA loudspeaker prototypes made of different combinations of active film and substrate materials, with a total surface area of 5.0 × 5.0 cm².

For the substrates, we considered both 1.5 mm thick soda-lime glass slabs and 75 um thick polyimide films (Kapton^®^ from DuPont, Wilmington, DE, USA). For the active film materials, we considered both thermally evaporated gold films and a spray-coated solution-based silver nanowires (AgNW) random network. The active film materials were contacted using copper tape and conductive silver ink, which leads to an active area of 3.5 × 5.0 cm².

The gold films were deposited by physical vapor deposition (PVD) with three thicknesses of 20, 40 and 100 nm, respectively, at a base pressure of 5 × 10^−6^ mbar and a deposition rate of 3 Å/s utilizing an UNIVEX 250 Leybold (Cologne, Germany). Figure 1b,c shows a TA loudspeaker sample with 100 nm thick gold active film on glass and Kapton, respectively.

Solution-based AgNW with a mean length and mean diameter of 25 μm and 40 nm, respectively, were diluted with isopropyl alcohol (5 wt.%) and spray-coated to transparent and conducting random networks, as described in [19]. The spraying that is referred to 1 layer in this study corresponds to the deposition of a wire network with a density of 0.1 wires/μm². Figure 1d,e shows a TA loudspeaker sample with 15 layers of AgNW active film on glass and Kapton, respectively. Figure 1f,g shows SEM-images of the AgNW random network deposited on glass and Kapton, respectively.

### 2.2. Electric Characterization

The electrical resistance of the fabricated TA loudspeakers was measured using a Jandel (Linslade, UK) 4-point probing system connected to a Keysight (Santa Rosa, CA, USA) B2901A source measuring unit. A constant DC current of 0.1 mA was sourced for all measurements.

### 2.3. Thermal Characterization

#### 2.3.1. Setup

**Figure 2:**
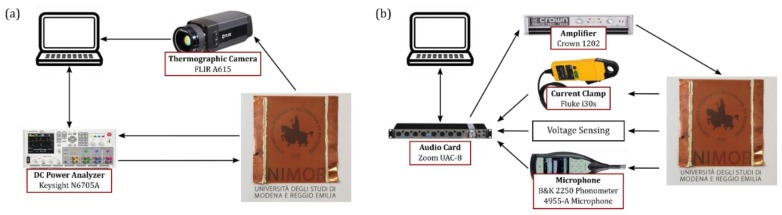
(**a**) The temperature measurement setup: a Keysight N6705A DC power analyzer is used to generate the input voltage signal, and simultaneously acquire the instantaneous voltage and current signals. The temperature data is acquired using a FLIR A615 thermographic camera; (**b**) The pressure measurement setup: A Zoom UAC-8 is used to generate and acquire signals. The input signals are amplified through a Crown 1202 amplifier. Voltage, current and pressure signals are conditioned and acquired.

The experimental setup for the thermal characterization of TA loudspeakers is depicted in Figure 2a. A Keysight (Santa Rosa, CA, USA) N6705A DC power analyzer is used to simultaneously generate the input signal and acquire the instantaneous voltage and current signals. The device is programmed and controlled via a MATLAB script. The temperature of the TA loudspeaker surface is measured using a FLIR (FLIR Systems, Wilsonville, OR, USA) A615 thermographic camera. The acquired electrical and thermal data are processed in MATLAB 2017a (The MathWorks, Natick, MA, USA) to derive the input power to temperature frequency response.

#### 2.3.2. Method

The thermal characterizations were performed using the Exponential Sine Sweep (ESS) method [20,21]. This technique allows the estimation of the impulse and frequency responses measuring the system response to an ESS, which is a sinusoidal signal with a frequency that increases exponentially with time. In this work, this method is used for the first time to measure the thermal frequency response of TA loudspeakers.

The application of the ESS method to thermal systems requires special care due to the unavoidable spurious transients and DC components, both in the input stimulus and in the output response. Such contributions are not considered in the ESS method and would produce errors in the response estimate if not properly accounted. 

**Figure 3:**
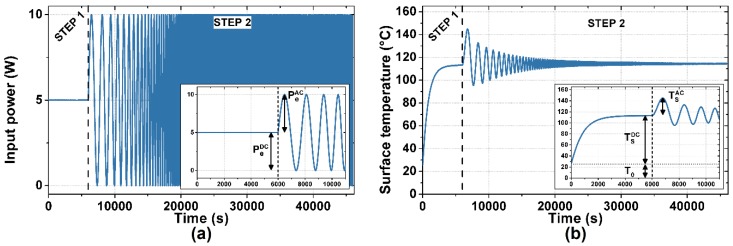
Applied power signal and measured temperature signal of a thermal measurement performed on samples with a glass substrate: (**a**) the input power signal, composed of a 6000 s long DC signal and a 40,000 s long biased Exponential Sine Sweep (ESS); (**b**) the measured temperature signal, with well separated transient and ESS response. The insets show a detail of the measured signals in the first 11,000 s.

The measurement procedure, designed accordingly and consisting of two steps as shown in Figure 3, is followed by post-processing. In the first step, a DC voltage is applied to the device under test to set the working point. The constant power P_e_^DC^ dissipated on the active film determines a thermal transient of the surface temperature. The duration of the first step is made long enough for the temperature to reach the DC steady state T_S_^DC^. In the second step, the ESS stimulus is applied. An ESS voltage is applied to the device under test, inducing an ESS instantaneous power dissipation P_e_^AC^ + P_e_^DC^, and thus a thermal response of its surface temperature T_S_^AC^ + T_S_^DC^. The DC component of the induced power dissipation P_e_^DC^ is kept constant throughout the two steps, preventing the occurrence of further thermal transients. The applied ESS voltage spans from 0.5 mHz to 1 Hz in 40,000 s, inducing a biased ESS power dissipation spanning from 1 mHz to 2 Hz with P_e_^DC^ = P_e_^AC^ = 5 W, as shown in Figure 3a.

The instantaneous voltage, current, and temperature signals are simultaneously acquired during the measurement. The voltage and current signals are used to calculate the instantaneous power dissipation, after being filtered out to avoid frequency aliasing. The power and temperature data are then post-processed, isolating the ESS-related data and removing the DC component. Finally, the thermal frequency response (power to temperature) is computed using the ESS method. 

### 2.4. Acoustic Characterization

#### 2.4.1. Setup

The experimental setup for the acoustic characterization of TA loudspeakers is depicted in Figure 2b. A Zoom UAC-8 sound card is used as I/O interface to manage input and output signals. The input signal is amplified using a Crown 1202 amplifier, while the instantaneous pressure, voltage, and current signals are simultaneously converted to proper voltage levels and acquired via the sound card. The pressure signal is measured using a Brüel & Kjær (Nærum, Denmark) 2250 phonometer equipped with a Brüel & Kjær (Nærum, Denmark) 4955-A microphone, calibrated using a Brüel & Kjær (Nærum, Denmark) 4231 sound calibrator (94 dB SPL reference). The voltage signal is conditioned through a buffered voltage divider. The current is measured using a Fluke i30s current clamp. MATLAB is used to control the audio card, generating a stimulus signal and simultaneously acquiring the conditioned signals, and to process the acquired data to derive the power to pressure frequency response. Data acquisition and post-processing have been realized by using the ITA-Toolbox [22]. The sample is mounted on an IEC compliant acoustic baffle [23], used to approximate the infinite baffle condition. All the acoustic measurements are performed inside an insulating anechoic box (1.4 × 1.5 × 1.7 m^3^). The microphone is placed on-axis, at 0.5 m distance from the TA loudspeaker surface, i.e., in far field.

#### 2.4.2. Method

The acoustic measurements were also performed using the ESS method. An ESS voltage, spanning from 50 Hz to 12000 Hz in 20 s, is applied to the device under test, inducing an ESS instantaneous power dissipation P_e_^DC^ + P_e_^AC^, spanning from 100 Hz to 24000 Hz with P_e_^DC^ = P_e_^AC^ = 20 W. The instantaneous voltage, current, and temperature signals are simultaneously acquired during the measurement. The voltage and current signals are used to calculate the instantaneous power dissipation, after being filtered out to avoid frequency aliasing. Then, the power data is post-processed, removing the DC component. Finally, the acoustic frequency response (power to pressure) is computed using the ESS method.

## 3. Results

The fabricated active films are characterized by moderate to low electrical resistance and very low heat capacity per unit area. Table 1 reports the values of electrical resistance (measured) and heat capacity per unit area (estimated) of the considered TA loudspeakers.

Interestingly, the AgNW active films are characterized by electrical resistance values comparable with the 20 nm thick solid gold films, despite their random network structure. Moreover, AgNW active films are expected to exhibit the lowest heat capacity per unit area among the considered samples. This is ascribed to the very low matter content of the random network structure.

**Figure 4:**
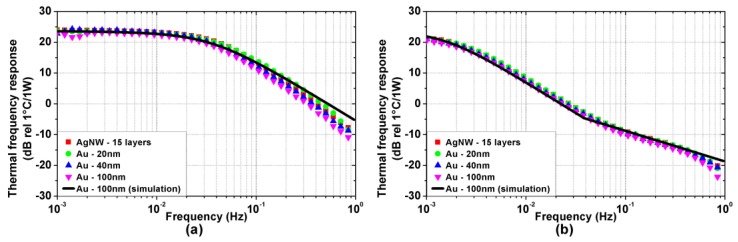
Experimental (colored symbols) and simulated (black solid line) thermal frequency responses (power to temperature) of the TA loudspeaker samples on (**a**) Kapton and (**b**) glass substrate.

Figure 4a,b show the measured thermal frequency responses of the TA devices on Kapton and glass substrate, respectively. The thermal frequency responses have low-pass behavior, in accordance with experimental results found in the literature [18]. Interestingly, the response DC value does not depend on specific TA loudspeaker properties, whereas the cut-off frequency is much higher for the Kapton substrate devices than for the glass ones, highlighting the strong influence of the substrate on the thermal dynamics. Furthermore, the devices with glass substrate exhibit a transition in the stopband slope, from −20 dB/dec to −10 dB/dec. This feature of the TA loudspeaker thermal response has never been reported before and can be clearly detected thanks to the ESS method. Finally, the material and the thickness of the active film appear not to influence the thermal response in any significant way.

**Figure 5:**
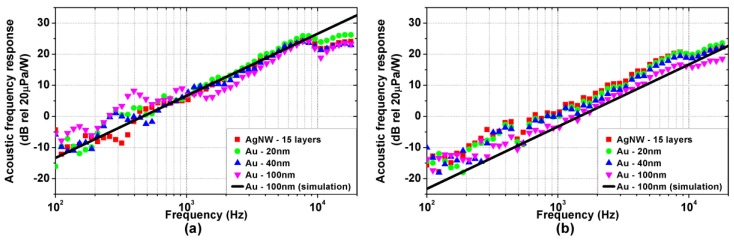
Experimental (coloured symbols) and simulated (black solid line) acoustic frequency responses (power to pressure) normalized at 1m distance of the TA loudspeaker samples: (**a**) Kapton substrate; (**b**) glass substrate.

Figure 5a,b show the measured acoustic frequency responses of the TA devices on Kapton and glass substrate, respectively, normalized at 1 m distance. As reported in the literature [2,14], a clear high-pass frequency trend is observed, with a constant +20 dB/dec slope. The overall acoustic response is mostly influenced by the substrate properties, with the higher magnitude observed on TA devices with the Kapton substrate. Again, the material and the thickness of the active film do not influence the acoustic response significantly.

## 4. Discussion

The presented experimental results prove that the thermal and the acoustic responses of TA loudspeakers are characterized by opposite frequency trends, i.e., low-pass and high-pass, respectively, which do not depend on specific device geometry or materials.

Moreover, as shown by in Figure 4 and Figure 5, the experimental results are accurately reproduced by the simulations performed using the model proposed in [19]. For sake of clarity, only the simulations for the 100 nm thick gold active film are reported, as no significant difference emerges in the measured bandwidth among the considered active films due to their extremely low heat capacity per unit area. The simulations are performed considering the parameters reported in Table 1 and Table 2. The heat transfer coefficients associated with the thermal losses of air and substrate are $h_{AIR}=h_{SUB}=13\frac{W}{m^{2}K}$, and the total heat transfer coefficient is $h_{TOT}=h_{AIR}+h_{SUB}=26\frac{W}{m^{2}K}$.

### 4.1. Thermal Behavior

The thermal behavior of the TA loudspeaker is investigated through the model presented in [18]:

(1) $$T_{S}^{AC}\left(j\omega \right)=P_{e}^{AC}\left(j\omega \right)\frac{1}{S}\frac{1}{j\omega (c_{S}+c_{SUB})+2\sqrt{j\omega }e_{AIR}+h_{TOT}}\;\mathrm{for}\;\omega <2\pi f_{T}$$

(2) $$T_{S}^{AC}\left(j\omega \right)=P_{e}^{AC}\left(j\omega \right)\frac{1}{S}\frac{1}{j\omega c_{S}+\sqrt{j\omega }\left(e_{AIR}+e_{SUB}\right)+h_{TOT}}\;\mathrm{for}\;\omega >2\pi f_{T}$$

where $c_{S}$ and $c_{SUB}$ are the heat capacity per unit area of the active film and the substrate, respectively, $e_{AIR}$ and $e_{SUB}$ are the thermal effusivity of the air and substrate, respectively, S is the surface area of the TA loudspeaker, and $f_{T}=\frac{\alpha_{SUB}}{2\pi L^{2}}$, with $\alpha_{SUB}$ being the substrate thermal diffusivity and L the substrate thickness, is the transition frequency at which the substrate switch from a capacitive to effusive behavior.

For $f<f_{T}$, the substrate temperature distribution is approximately uniform and thus the substrate can be effectively modeled as a heat capacity. The TA loudspeaker thermal response is dominated by the thermal losses of air and substrate, and by the heat capacity of the substrate, which is much larger than the one of the active film. This results in a first-order low-pass dynamic with cut-off frequency $f_{C}=\frac{h_{TOT}}{2\pi c_{SUB}}$. The calculated cut-off frequencies, which are $f_{C}=17.8\times 10^{-3}\;Hz$ and $f_{C}=0.74\times 10^{-3}\;Hz$ for the Kapton and glass substrate, respectively, are in agreement with the experimental results shown in Figure 4. 

For $f>f_{T}$ the substrate temperature distribution exhibits a fast exponential decay along its thickness, preventing any thermal fluctuation to reach the back of the substrate. From a thermal perspective, the substrate can be approximated as a semi-infinite medium, characterized by a thermal effusivity $e_{SUB}$. The thermal response, while retaining a low pass dynamic, exhibits a slope change as observed in Figure 4b at $f_{T}=37.7\times 10^{-3}\;Hz$ for the glass substrates, whereas it is not visible in Figure 4a since $f_{T}=2.2\;Hz$ for the Kapton substrates.

**Figure 6:**
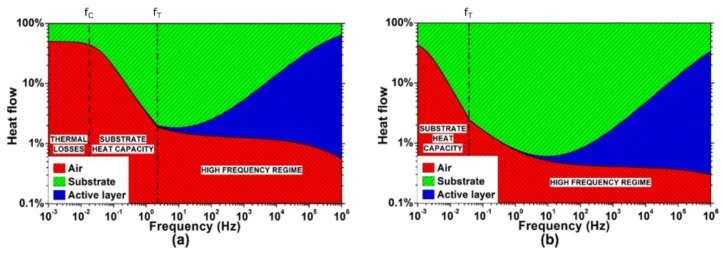
Simulated distribution of the heat flows in the TA loudspeaker: (**a**) Kapton substrate; (**b**) glass substrate. Logarithmic scale has been used to better visualize the fraction of heat flow injected into the air.

A better insight into the thermal interactions taking place during the TA loudspeaker operation requires the analysis of the three involved heat flows: the one injected into the air ${\dot{Q}}_{AIR}$, the one injected into the substrate ${\dot{Q}}_{SUB}$, and the one drained by the active film heat capacity ${\dot{Q}}_{AL}$. Figure 6 shows the simulations of the heat flows distribution generated in a TA loudspeaker with 100 nm thick gold active film on both Kapton and glass substrates. The parameters are the same considered for the simulation of the thermal responses of Figure 4. Interestingly, the heat flows distribution is initially dominated by the substrate-air interaction, while the effects of the active film arise only at a higher frequency.

For $f<f_{T}$, the three heat flows simplify as follows, provided that the heat capacity of the substrate is much larger than the active film one ($c_{SUB}+c_{AL}\approx c_{SUB})$ and the small contribution of the air thermal effusivity ($\sqrt{j\omega }e_{AIR}\to 0$) is neglected:

(3) $${\dot{Q}}_{AIR}\left(j\omega \right)\approx P_{e}^{AC}\left(j\omega \right)\frac{h_{AIR}}{j\omega c_{SUB}+h_{TOT}}$$

(4) $${\dot{Q}}_{SUB}\left(j\omega \right)\approx P_{e}^{AC}\left(j\omega \right)\frac{j\omega c_{SUB}+h_{SUB}}{j\omega c_{SUB}+h_{TOT}}$$

(5) $${\dot{Q}}_{AL}\left(j\omega \right)\approx 0$$

Equations (3)–(5) show that the input heat flow mostly divides between air and substrate. Above the cut-off frequency $f_{C}$, the heat capacity of the substrate increasingly drains the input heat flow, greatly limiting the heat injection into the air. The active film, having a negligible heat capacity, drains almost no heat.

The heat capacity of the substrate dominates the TA loudspeaker thermal dynamic until $f>f_{T}$, when the substrate assumes an effusive behavior, allowing for the emergence of otherwise negligible thermal interactions. The resulting heat flows are the following:

(6) $${\dot{Q}}_{AIR}\left(j\omega \right)=P_{e}^{AC}\left(j\omega \right)\frac{\sqrt{j\omega }e_{AIR}}{j\omega c_{S}+\sqrt{j\omega }\left(e_{AIR}+e_{SUB}\right)+h_{TOT}}$$

(7) $${\dot{Q}}_{SUB}\left(j\omega \right)=P_{e}^{AC}\left(j\omega \right)\frac{\sqrt{j\omega }e_{SUB}}{j\omega c_{S}+\sqrt{j\omega }\left(e_{AIR}+e_{SUB}\right)+h_{TOT}}$$

(8) $${\dot{Q}}_{AL}\left(j\omega \right)=P_{e}^{AC}\left(j\omega \right)\frac{j\omega c_{S}}{j\omega c_{S}+\sqrt{j\omega }\left(e_{AIR}+e_{SUB}\right)+h_{TOT}}$$

where $e_{AIR}$ is the air thermal effusivity, $e_{SUB}$ is the substrate thermal effusivity, and $c_{S}$ is the active film heat capacity per unit area. 

The TA loudspeaker thermal behavior is determined by the interactions between the substrate and air thermal effusivities, and the heat capacity of the active film. As shown in Equation (6), the heat flow injected into the air ${\dot{Q}}_{AIR}$, which is the fraction involved in the thermoacoustic transduction, is characterized by a band-pass dynamic.

While the lower cut-off frequency $f_{L}\approx \frac{1}{2\pi }{\left(\frac{h_{TOT}}{e_{AIR}+e_{SUB}}\right)}^{2}$ can be neglected being typically $f_{L}<f_{T}$, the higher cut-off frequency $f_{H}\approx \frac{1}{2\pi }{\left(\frac{e_{AIR}+e_{SUB}}{c_{S}}\right)}^{2}$ is of paramount importance, as it defines the frequency at which the active film heat capacity starts to affect the TA loudspeaker, draining the input heat flow and preventing it to be injected into the air. In fact, the heat drained by the active film ${\dot{Q}}_{AL}$ exhibits a high-pass behavior. 

In the pass-band region, the input heat flow divides between air and substrate, according to the effusivity ratio $\frac{e_{AIR}}{e_{AIR}+e_{SUB}}$. As also reported in [13], the effusivity ratio determines the maximum fraction of the input heat flow that can be transferred to the air by conduction. Noticeably, for the considered devices, the fraction of the input heat flow injected into the air and converted into sound in the audio bandwidth (e.g., within 20 Hz and 20 kHz) is 0.4% for the glass and 1.4% for the Kapton, while the rest is drained by the substrate. 

This explains the lower efficiency of the considered TA loudspeakers, characterized by a solid substrate, with respect to the suspended ones. Nevertheless, the heat injection into the air can be significantly improved increasing the effusivity ratio, i.e., using substrates characterized by low thermal effusivity.

### 4.2. Acoustic Behavior

As explained in [18], a direct relation exists between the heat flow injected into the air ${\dot{Q}}_{AIR}$, and the generated far field pressure $p_{FF}$:

(9) $$p_{FF}\left(r,\omega \right)=j\omega P_{e}^{AC}\left(j\omega \right)\frac{\gamma -1}{c_{0}^{2}}\frac{\sqrt{j\omega }e_{AIR}}{j\omega c_{S}+\sqrt{j\omega }\left(e_{AIR}+e_{SUB}\right)+h_{TOT}}\frac{e^{-\frac{j\omega }{c_{0}}r}}{2\pi r}=j\omega {\dot{Q}}_{AIR}\left(j\omega \right)\frac{\alpha_{V}}{c_{P}}\frac{e^{-\frac{j\omega }{c_{0}}r}}{2\pi r}$$

where $\alpha_{V}$ is the air volumetric thermal expansion coefficient, and $c_{P}$ is the air specific heat, γ is the air adiabatic index, $c_{0}$ is the adiabatic speed of sound in the air, and r is the on-axis distance from the TA loudspeaker surface.

The far-field pressure $p_{FF}$ depends on the time derivative of ${\dot{Q}}_{AIR}$, which sets the high-pass trend of the acoustic response. Consistently, as shown in Figure 5, the acoustic response has a +20 dB/dec frequency trend within the band-pass region of ${\dot{Q}}_{AIR}$. Moreover, the pressure response is scaled accordingly to the distance r and the air TA coefficient $\frac{\alpha_{V}}{c_{P}}$. The latter, as also reported in previous studies [15], relates the heat flow injected into the air to the induced air mass flow due to thermal expansion, which is key for the generation of the pressure wave [24].

The extremely low value of the air TA coefficient ($\frac{\alpha_{V}}{c_{P}}\approx 3.3\times 10^{-6}\;\frac{kg}{W\cdot s}$) highlights the weak coupling between the thermal and the acoustic domain in the air, which can be recognized as the main cause for the low efficiency of the TA sound generation. Studies on the thermoacoustic transduction in different fluids [25,26] confirmed the influence of the TA coefficient, reporting improvements using low specific heat gases (e.g., Argon).

Interestingly, the thermoacoustic transduction described in Equation (7) does not introduce any high-frequency cut-off, nor is it affected by the geometry or the materials of the TA loudspeaker. The cut-off phenomena reported in the literature [1,11,16], as well as the dependence of the generated pressure on the substrate material shown in Figure 5, are to be ascribed to the thermal interactions discussed in Section 4.1, and not to the thermoacoustic transduction per se. 

Indeed, the pressure high-frequency cut-off is caused by the heat flow drain of the active film, limiting the heat injection into the air at high frequency. The dependence of the pressure response on the substrate material, instead, is due to the effusivity ratio affecting the band-pass heat injection into the air.

## 5. Conclusions

We have investigated the opposite frequency trends shown by the thermal and the acoustic responses of nanostructured TA loudspeakers. The experimental characterization of both thermal and acoustic responses of such devices has been interpreted using a physical model. We have found that the low-pass trend of the thermal response is mostly determined by the substrate, behaving as a heat capacity at low frequencies. Nevertheless, thanks to the substrate transition to an effusive behavior, a flat heat injection into the air is enabled at high frequency. The high-pass pressure response is recognized as the result of the TA effect taking place in the air when injected with such flat heat flow. The reported influence of the substrate and the active film on the pressure response are now recognized as arising from the underlying thermal interactions.

## Figures and Tables

**Table 1:** Properties of the thermoacoustic loudspeaker samples.

Substrate	Active Film	Electrical Resistance *R_e_* (Ω)	Heat Capacity Per Unit Area $c_{S}\;\left(\frac{J}{m^{2}\cdot K}\right)$
Kapton—75 μm	AgNW—15 layers	3.82	39.3 × 10^−3^ [19]
Kapton—75 μm	Gold—20 nm	2.51	50.2 × 10^−3^
Kapton—75 μm	Gold—40 nm	1.23	100.5 × 10^−3^
Kapton—75 μm	Gold—100 nm	0.39	251.2 × 10^−3^
Glass—1.5 mm	AgNW—15 layers	2.92	39.3 × 10^−3^ [19]
Glass—1.5 mm	Gold—20 nm	2.54	50.2 × 10^−3^
Glass—1.5 mm	Gold—40 nm	0.94	100.5 × 10^−3^
Glass—1.5 mm	Gold—100 nm	0.36	251.2 × 10^−3^

**Table 2:** Thermophysical properties of the materials.

Property	Symbol (Unit)	Air	Kapton	Glass
Thermal conductivity	$\kappa \;\left(\frac{W}{m\cdot K}\right)$	0.0263	0.12	1
Density	$\rho \;\left(\frac{\mathrm{kg}}{m^{3}}\right)$	1.16	1420	2500
Specific heat	$c_{P}\;\left(\frac{J}{\mathrm{kg}\cdot K}\right)$	1007	1090	750
Thermal diffusivity	$\alpha \;\left(\frac{m^{2}}{s}\right)$	22.6 × 10^−6^	77.5 × 10^−9^	533.3 × 10^−9^
Thermal effusivity	$e\;\left(\frac{W\cdot s^{0.5}}{m^{2}\cdot K}\right)$	5.5	431	1369.3
